# *Amorphophallus muelleri* activates ferulic acid and phenylpropane biosynthesis pathways to defend against *Fusarium solani* infection

**DOI:** 10.3389/fpls.2023.1207970

**Published:** 2023-07-05

**Authors:** Penghua Gao, Ying Qi, Lifang Li, Shaowu Yang, Jiani Liu, Huanyu Wei, Feiyan Huang, Lei Yu

**Affiliations:** College of Agronomy, Yunnan Urban Agricultural Engineering and Technological Research Center, Kunming University, Kunming, China

**Keywords:** konjac, phenylpropane biosynthesis, plant-pathogen interaction, resistance genes, *Fusarium solani*

## Abstract

*Amorphophallus* sp. is an economically important crop for rural revitalization in southwest China. However, *Fusarium solani* often infects *Amorphophallus* sp. corms during storage, damaging the corm quality and affecting leaf elongation and flowering in the subsequent crop. In this study, the mechanism of resistance to *F. solani* was investigated in the leaf bud and flower bud corms of *Amorphophallus muelleri* through transcriptome and metabolome analyses. A total of 42.52 Gb clean reads and 1,525 metabolites were detected in a total of 12 samples including 3 samples each of disease-free leaf bud corms (LC), leaf bud corms inoculated with *F. solani* for three days (LD), disease-free flower bud corms (FC), and flower bud corms inoculated with *F. solani* for three days (FD). Transcriptome, metabolome, and conjoint analyses showed that ‘MAPK signal transduction’, ‘plant-pathogen interaction’, ‘plant hormone signal transduction’, and other secondary metabolite biosynthesis pathways, including ‘phenylpropane biosynthesis’, ‘arachidonic acid metabolism’, ‘stilbene, diarylheptane and gingerolin biosynthesis’, and ‘isoquinoline alkaloids biosynthesis’, among others, were involved in the defense response of *A. muelleri* to *F. solani*. Ultimately, the expression of six genes of interest (*AmCDPK20*, *AmRBOH*, *AmWRKY33*, *Am4CL*, *Am* POD and *AmCYP73A1*) was validated by real-time fluorescence quantitative polymerase chain reaction, and the results indicated that these genes were involved in the response of *A. muelleri* to *F. solani*. Ferulic acid inhibited the growth of *F. solani*, reducing the harm caused by *F. solani* to *A. muelleri* corms to a certain extent. Overall, this study lays a strong foundation for further investigation of the interaction between *A. muelleri* and *F. solani*, and provides a list of genes for the future breeding of *F. solani*-resistant *A. muelleri* cultivars.

## 1 Introduction

*Amorphophallus muelleri* is widely planted in Maitreya and Xishuangbanna in the Yunnan Province of China, because of its strong soft-rot resistance, high yield, and high glucomannan content. However, *A. muelleri* corms are often infected with *Fusarium solani*, which causes corm and petiole rot.

*F. solani* has a wide host range and can infect many important agricultural crops such as potato (*Solanum tuberosum*), *Gastrodia elata*, and soybean (*Glycine max*) (Gherbawy et al., 2021; Li and Cheng, 2022; Yan and Nelson, 2022). *F. solani* usually infects the root or stem of the host plant, causing rot, stunting, and wilting, and the degree of necrosis correlates with the severity of the disease (Coleman, 2016).

To minimize the damage caused by pathogen infection, host plants alter gene expression levels, which changes the metabolite levels. The combination of transcriptomic and metabolomic analyses is widely used to screen genes and metabolites involved in plant disease resistance. Zhu et al. (2022) analyzed the transcriptome and metabolome data of two soybean genotypes infected with *Colletotrichum truncatum*, and found that jasmonic acid and auxin synthesis, mitogen-activated protein kinase (MAPK) and Ca^2+^signaling, *WRKY* and *bHLH* transcription factors, disease resistance related genes, and terpenoid metabolites are involved in the resistance of soybean to *C. truncatum*. Similarly, transcriptome and metabolome data analyses of sorghum (*Sorghum bicolor*) plants infected with *Puccinia sorghi* showed induction of the expression of phenylpropanoid, flavonoid, and terpenoid metabolic pathway genes and increase in the content of intermediate metabolites (Kim et al., 2021). Thus, the above studies show that conjoint multi-omics analysis has become an important way to explore plant disease resistance mechanisms.

The phenylpropane biosynthesis pathway is involved in the synthesis of lignin, metabolization of various compounds such as ferulic acid (FA) and P-coumaric acid, and production of the plant disease resistance hormone salicylic acid, which is involved in pathogen resistance. In *Bambusa pervariabilis × Dendrocalamopsis grandis*, *CCoAOMT2* and *CAD5* genes participate in the resistance to shoot blight, caused by *Arthrinium phaeospermum*, by regulating the synthesis of lignin and flavonoids (Luo et al., 2022). In the resistant wheat (*Triticum aestivum*) cultivar ‘H83’, the phenylpropanoid biosynthesis pathway is specifically activated after infection with *Rhizoctonia cerealis* (Geng et al., 2022). These studies suggest that phenylpropanoid biosynthesis genes and metabolites are involved in plant resistance to pathogens.

To date, only a few studies have been conducted on the resistance of *A. muelleri* to *F. solani*. Therefore, in this study, we aimed to determine the theoretical basis of *F. solani* resistance in *A. muelleri*. Specifically, we analyzed changes in gene expression and metabolite content in the flower bud and leaf bud corms of *A. muelleri* at an early stage of *F. solani* infection.

## 2 Materials and methods

### 2.1 Plant growth and plant infection

Forty flower bud corms and forty leaf bud corms of disease-free *A. muelleri* were selected. All corms were stored under controlled conditions (27 ± 2 °C, 16-h light/8-h dark photoperiod, and ~80% relative humidity).

*A. muelleri* corms with typical symptoms were collected, and isolates were obtained from the samples by culturing on potato dextrose agar (PDA; 40 g per L of dH_2_O) medium at 27 °C. Then, marginal hyphae were selected to obtain purified colonies and strains for subsequent experiments. Fungal species were identified based on the morphological evaluation of PDA plates and on the sequencing of ITS and *EF1* gene fragments. To conduct the pathogenicity test, *F. solani* was grown on PDA medium at 27 °C for 15 days. Spore inoculum was prepared by harvesting the spores in sterile water, filtering the spores through glass wool to remove the hyphae, and suspending the filtrate in potato dextrose broth (PDB; 18 g per L of dH_2_O) at a concentration of 10^5^ conidia mL^-1^. Subsequently, *A. muelleri* corms (*n* = 20) were injected with 100 µL of *F. solani* spore suspension with a sterile syringe. In the control treatment, *A. muelleri* corms (*n* = 10) were injected with sterile PDB.

To understand the defense response of *A. muelleri* leaf and flower corms to *F. solani* at an early stage of infection, the changes in gene expression and metabolite contents in infected leaf and flower corms were detected at 3 days post-inoculation (dpi) by transcriptome sequencing (three replicates) and metabolite determination (six replicates), respectively, with three corms per treatment.

Infected and control corms were harvested in a randomized manner. An area of the corm located 3 cm away from the injection site was sampled, ground into a fine powder using liquid nitrogen, and stored at -80 °C until needed for subsequent analysis.

### 2.2 RNA extraction, library construction, and sequencing

Total RNA was extracted from the flower bud and leaf bud corms of *A. muelleri* using the TRIzol Reagent (Invitrogen, Carlsbad, CA, USA), according to the manufacturer’s instructions. The quality of the total RNA was checked by agarose gel electrophoresis and using Agilent 2100 Bioanalyzer (Agilent Technologies, Palo Alto, CA, USA). Subsequently, the extracted total RNA was enriched by oligo(dT) beads. The enriched mRNA was fragmented into short fragments using fragmentation buffer, and reverse transcribed into double-stranded cDNA. Then, the cDNA was purified, end-repaired, adenylated, and ligated to Illumina sequencing adapters. Subsequently, the cDNA from the previous study was purified. Fragments of the appropriate size were selected by polymerase chain reaction (PCR) and agarose gel electrophoresis. Finally, the resulting cDNA library was sequenced on the Illumina NovaSeq6000 platform by Gene Denovo Biotechnology Co. (Guangzhou, China).

### 2.3 RNA-Seq data analysis

Firstly, the RNA-Seq reads containing adapters, more than 10% of unknown nucleotides (Ns), and more than 50% of low-quality bases (Q-value ≤ 20) were removed by fastp (version 0.18.0) (Chen et al., 2018). The resultant high-quality cleans reads were mapped to the ribosome RNA (rRNA) database using the short read alignment tool Bowtie2 (version 2.2.8) (Langmead and Salzberg, 2012). Then, the reads mapped to rRNAs were removed, and the remaining clean reads were used for assembly and analysis. Clean paired-end reads were mapped on to the reference genome using HISAT2. 2.4, with ‘-rna-strandness RF’ and default settings for all other parameters (Pertea et al., 2016). The mapped reads belonging to 12 samples, including 3 samples each of disease-free leaf bud corms (LC), *F. solani*-inoculated leaf bud corms collected at 3 dpi (LD), disease-free flower bud corms (FC), and *F. solani*-inoculated flower bud corms collected at 3 dpi (FD), were assembled using StringTie v1.3.1 (Pertea et al., 2015). Then, a fragment per kilobase of transcript per million mapped reads (FPKM) value was calculated for all samples, and variation in gene expression levels was determined using the RNA-Seq by Expectation Maximization (RSEM) software (Li and Dewey, 2011). Differential gene expression analysis was conducted on the four groups using the DESeq2 software (Love et al., 2014), and genes with a false discovery rate (FDR) of <0.05 and absolute fold change of ≥2 were designated as differentially expressed genes (DEGs). Finally, the DEGs were functionally annotated using Gene Ontology (GO) (Ashburner et al., 2000) and Kyoto Encyclopedia of Genes and Genomes (KEGG) (Ogata et al., 1999) enrichment analyses.

### 2.4 Metabolite extraction and quantification

The flower bud and leaf bud corms were frozen in liquid nitrogen immediately after harvesting and ground into powder. Then, 1 mL of methanol: acetonitrile: H2O (2:2:1, v/v/v) was added for metabolite extraction. The extracted metabolites were analyzed by Shanghai Applied Protein Technology Co., Ltd using an ultra-high perform liquid chromatography (UHPLC) system (1290 Infinity LC, Agilent Technologies) coupled to a quadrupole time-of-flight (AB Sciex TripleTOF 6600). A quality control (QC) sample was prepared by mixing equal volumes of all 12 samples to be tested. To perform hydrophilic interaction liquid chromatography (HILIC), the 12 samples were analyzed using a separation column (2.1 mm × 100 mm ACQUIY UPLC BEH 1.7 µm column; Waters, Ireland). Solvent A (25 mM ammonium acetate and 25 mM ammonium hydroxide in water) and solvent B (acetonitrile) were used as the mobile phase. The following gradient applied: 95% B for 0.5 min; linear reduction to 65% B in 6 min; further reduction to 40% B in 0.1 min, followed by a hold for 8 min; increase to 95% B in 0.1 min, followed by a 3-min re-equilibration period. The primary and secondary spectra of 12 samples were collected using an AB Triple TOF 6600 mass spectrometer. The ESI source conditions after HILIC chromatographic separation were as follows: ion source gas1 (Gas1): 60; ion source gas2 (Gas2): 60; curtain gas (CUR): 30; source temperature: 600 °C; ion sapary voltage floating (ISVF): ± 5500 V; TOF MS scan m/z range: 60–1000 Da; product ion scan m/z range: 25–1000 Da; TOF MS scan accumulation time 0.20 s per spectrum; product ion scan accumulation time: 0.05 s per spectrum. The secondary mass spectrum was obtained through information-dependent acquisition (IDA), with high sensitivity mode. The separation was as follows: collision energy (CE): fixed at 35 V, with ±15 eV; declustering potential (DP): 60 V (+) and −60 V (−); isotopes excluded within 4 Da; candidate ions monitored per cycle: 10.

### 2.5 Metabolomic data analysis

A variable importance in projection (VIP) score of orthogonal projection to latent structures (OPLS) model and *t*-test were used to screen differential metabolites. Metabolites with a *t*-test *p*-value < 0.05 and threshold VIP ≥ 1 were considered differentially accumulated metabolites between two groups. KEGG pathway enrichment analysis was conducted to further understand the characteristics of differentially accumulated metabolites (DAMs) (Kanehisa and Goto, 2000).

### 2.6 Combined metabolomic and transcriptomic analysis

To explain the resistance mechanism employed by *A. muelleri* corms against *F. solani*, a joint analysis of DEGs and DAMs was conducted based on the pathway model, a Two-way Orthogonal PLS (O2PLS) model (Bouhaddani et al., 2016), and the Pearson model.

### 2.7 Validation of RNA-Seq data

To validate the RNA-Seq data, the expression of six DEGs was examined by real-time fluorescence quantitative PCR (qRT-PCR) (**Supplemental Table S1**). Total RNA extracted from the FC, FD, LC, and LD samples was used to synthesize cDNA, which was then quantified on the Step OnePlus Real-Time Fluorescent Quantitative PCR system using 2×T5 Fast qRT-PCR Mix (SYBR Green I, China). Assays of each gene were repeated three times. The *actin* gene was used as an internal control. The quantification was evaluated using the 2^−(ΔΔCt)^ method.

### 2.8 Antifungal tests

Antifungal activity of FA was evaluated by inoculating 3 μL of the conidial suspension (1 × 10^5^ conidia per mL) on a 5-mm sterile filter paper disk placed in the center of a PDA plate containing increasing concentrations of FA (1, 20, 100 μg mL^-1^). The PDA plates were incubated at 28 °C. Colony growth was observed on the 3th, 5th and 7th day, the colony diameter of each group was measured with a vernier caliper, and the inhibition rate of strain growth was calculated. FA was dissolved in dimethyl sulfoxide (DMSO) for antifungal assays, and the maximum solvent amount for DMSO was 0.1% (v/v).

### 2.9 Exogenous FA treatment of *Amorphophallus muelleri* corms

Disease-free corms were harvested, cut into thin slices (~1 cm thick, 7.5 cm long, and 4 cm wide), and placed in a culture dish lined with a moistened sterile filter paper at the bottom. *F. solani* was grown in a PDA culture dish for 5 days, and mycelial discs were obtained using a 6-mm puncher. The mycelial side was placed on the corm slices, and the diameter of the disease spot was observed after 3, 4, and 5 days of inoculation. Corm slices sprayed with water served as the control group, while those sprayed with 100 μg mL^-1^ FA served as the experimental group. Each treatment was replicated three times.

## 3 Results

### 3.1 Identification of pathogenic fungi

**Figure 1:**
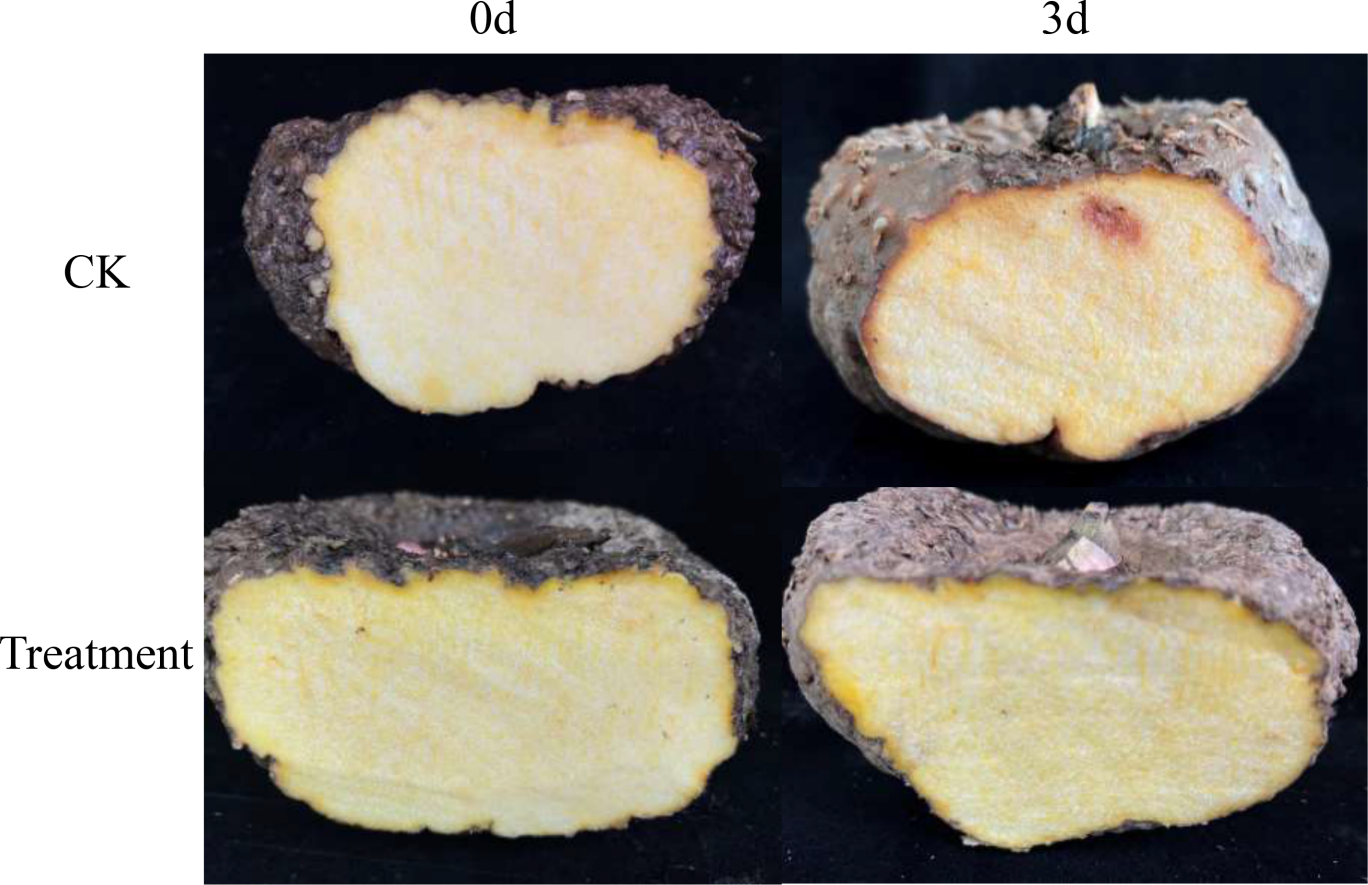
Symptoms of *Amorphophallus muelleri* corms after three days of inoculation with *F. solani*.

Morphological observation of the PDA plates showed dense, white mycelia. The small conidia were kidney-shaped or ovoid, and large conidia were nearly sickle-shaped and had blunt rounded tips (**Supplemental Figure S1**). The *EF1* (GenBank accession no. OR073911; 239 bp) and ITS (GenBank accession no. OP604148.1; 560 bp) sequences of the isolated fungi were 97.81% and 99.81% identical to those of *F. solani* isolates (GenBank accession nos. MH817435.1 and OQ318496.1, respectively). Based on these results, the fungus was identified as *F. solani.* In the pathogenicity test, all inoculated corms showed symptoms similar to those observed in the field, while the control corms showed no symptoms (**Figure 1**). The morphological and molecular identification of strains reisolated from the infected petioles fulfilled Koch’s postulates.

### 3.2 Overview of transcriptome analysis

Approximately 42.52 Gb data (PRJNA932136) were generated from a total of 12 samples (3 LC, 3LD, 3 FC, and 3 FD). The percentage of clean reads with Q > 20 and Q > 30 was approximately 97.46% and 93.06%, respectively, and the GC content varied between 47.89% and 49.52%, indicating that the sequenced fragments were of high quality (**Supplemental Table S2**). Principal component analysis (PCA) of these samples showed that all replicates exhibited similar expression patterns (**Supplemental Figure S2**), indicating that the RNA-Seq data were highly reliable. These results indicated that the RNA-Seq data were suitable for subsequent analysis.

**Figure 2:**
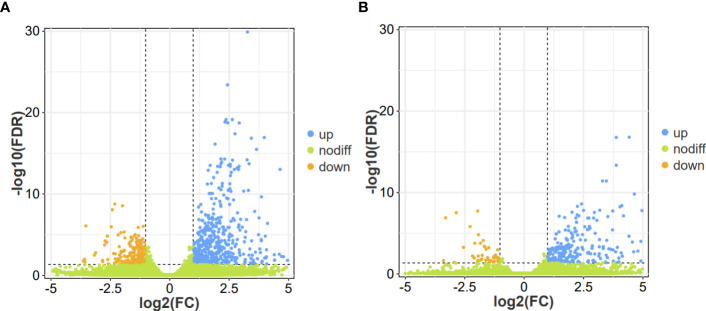
Volcano plot of differentially expressed genes (DEGs) identified in the leaf bud and flower bud corms of *A*. *muelleri* infected with *F*. *solani*. **(A, B)** Leaf bud corms **(A)** and flower bud corms **(B)** of *A*. *muelleri* at 3 d after *F*. *solani* infection.

To conduct a comprehensive overview of the gene expression profiles associated with the response of flower bud corms and leaf bud corms of *A. muelleri* to *F. solani* infection, DEGs was identified by using the DESeq2 software, based on two criteria: corrected *P*-value < 0.05, and |log2FC| ≥ 1. A total of 306 (262 upregulated, 44 downregulated) and 749 (547 upregulated, 202 downregulated) DEGs were found in the FC vs FD and LC vs LD comparisons, respectively (**Figure 2**). In these two comparisons, the most highly expressed genes included those encoding WRKY transcription factors, calcium-binding proteins, calcium-dependent protein kinases, mitogen-activated protein kinases (MAPKs), and plant hormone signaling factors, among others. This result indicated that *F. solani* infection altered gene expression in the flower bud and leaf bud corms of *A. muelleri.*

**Figure 3:**
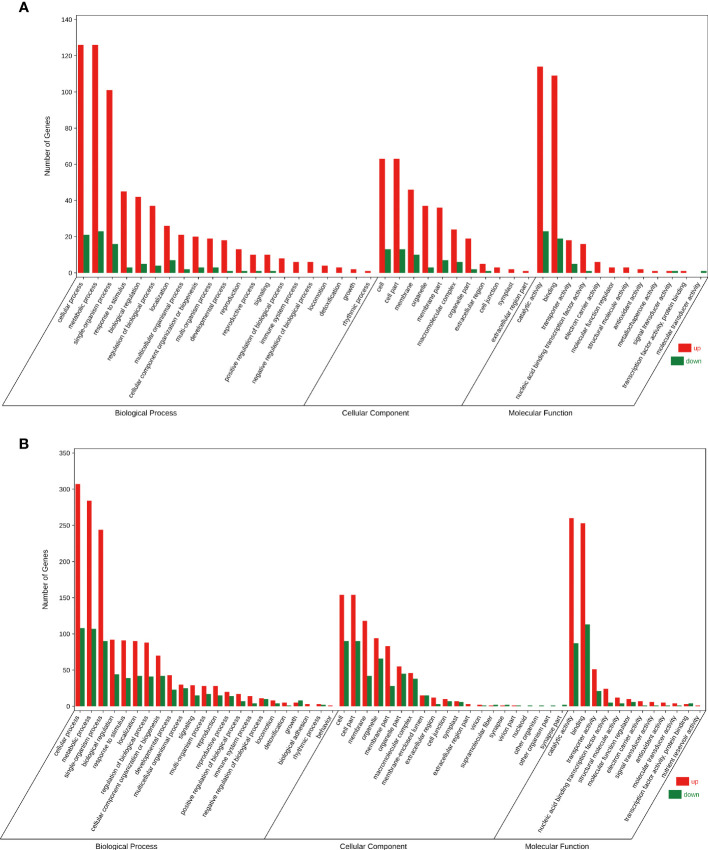
Gene Ontology bubble diagram of DEGs identified in the *F*. *solani*-infected leaf bud and flower bud corms of *A*. *muelleri*. **(A, B)** Leaf bud corms **(A)** and flower bud corms **(B)** of *A*. *muelleri* at 3 d after *F*. *solani* infection.

To understand the attributes of DEGs and their products, GO analysis was conducted using the GOseq software. In the FC vs FD and LC vs LD comparisons, the DEGs were mainly enriched in GO terms such as ‘cellular process’, ‘metabolic process’, and ‘single-organism process’ in the biological process category; ‘cell’, ‘cell part’, and ‘membrane’ in the cellular component category; and ‘catalytic activity’ and ‘binding’ in the molecular function category (**Figures 3A, B**). These results indicate that *F. solani* infection enhanced metabolic activity in *A. muelleri* corms.

**Figure 4:**
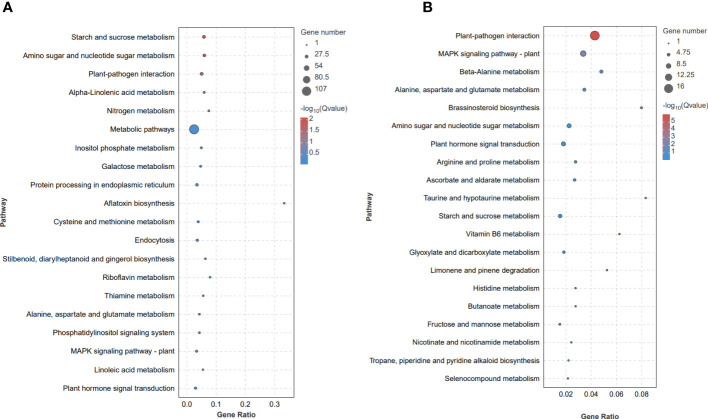
Kyoto Encyclopedia of Genes and Genomes (KEGG) bubble diagram of DEGs identified in the *F*. *solani*-infected leaf bud and flower bud corms of *A*. *muelleri*. **(A, B)** Leaf bud corms **(A)** and flower bud corms **(B)** of *A*. *muelleri* at 3 d after *F*. *solani* infection.

To further explore the main pathways activated by *F. solani* infection, a KEGG enrichment analysis of the DEGs was conducted. Of the 749 DEGs identified in the LC vs LD comparison, 196 DEGs were assigned to 81 KEGG pathways; these included genes involved in ‘starch and sucrose metabolism’, ‘amino sugar and nucleotide sugar metabolism’, ‘plant-pathogen interaction’, and ‘alpha-linolenic acid metabolism’, among other pathways (**Figure 4A**). Of the 306 DEGs identified in the FC vs FD comparison, 69 DEGs were assigned to 54 KEGG pathways; these included genes involved in ‘plant-pathogen interaction’, ‘MAPK signaling pathway-plant’, ‘beta-Alanine metabolism’, and ‘alanine, aspartate and glutamate metabolism’, among other pathways (**Figure 4B**). Taken together, these results showed that molecular signaling processes, amino acid metabolism, and plant hormone synthesis and signal transduction are activated in *A. muelleri* corms to induce resistance against *F. solani*.

**Figure 5:**
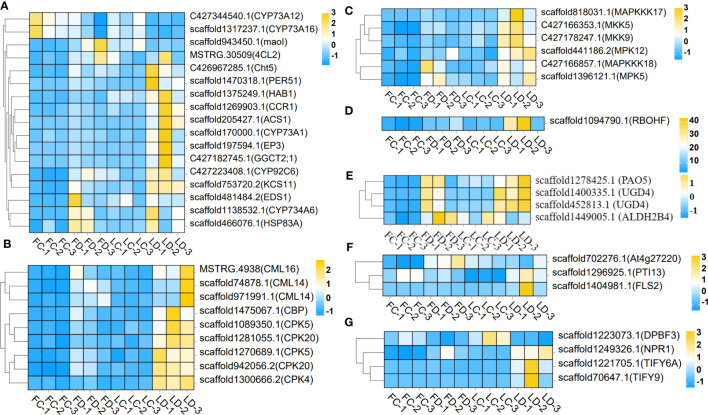
Heatmap of gene expression levels in the leaf bud corms and flower bud corms of A. muelleri infected with F. solani. The scale bar represents the expression level of each gene (FPKM) in different treatments, as indicated by yellow/blue rectangles. Genes in yellow were upregulated, and those in blue were downregulated. **(A)** Plant secondary metabolite biosynthesis pathway genes; **(B)** Ca^2+^ signaling pathway genes; **(C)** MAPK signaling pathway genes; **(D)** ROS metabolic pathway gene; **(E)** Ascorbic acid metabolism pathway genes; **(F)** PTI genes; **(G)** plant hormone signaling transduction pathway genes.

Heatmaps of DEGs subclusters were developed to better understand the key genes associated with *F. solani* resistance in *A. muelleri.* The results revealed DEGs involved in *A. muelleri*–*F. solani* interactions. Functional annotation analyses showed that these genes included three Ca^2+^-binding protein genes, six Ca^2+^-dependent protein kinase genes, six mitogen-activated protein kinase kinase genes, five antioxidant stress-related genes (including four ascorbic acid metabolism pathway genes and one respiratory burst oxidase gene), seventeen secondary metabolite biosynthesis pathway genes, disease resistance related genes, and four plant hormone (jasmonic acid, salicylic acid, and abscisic acid) signal transduction pathway genes (**Figure 5**). These findings suggest that *A. muelleri* corms respond to *F. solani* infection by activating the expression of genes involved in disease resistance pathways, plant hormone biosynthesis, anti-oxidation, and disease resistance compound biosynthesis.

### 3.3 Overview of metabolome analysis

**Figure 6:**
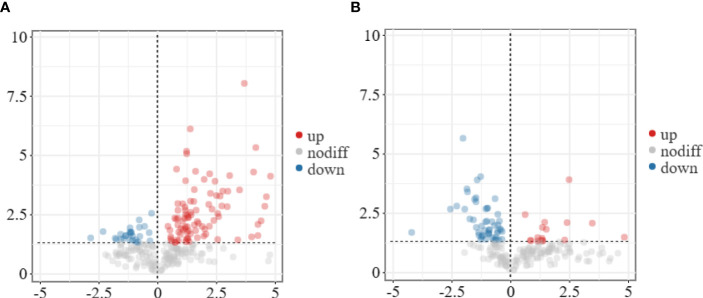
Volcano plot of DAMs identified in the leaf bud and flower bud corms of *A*. *muelleri* infected with *F*. *solani*. **(A, B)** Leaf bud corms **(A)** and flower bud corms **(B)** of *A*. *muelleri* at 3 d after *F. solani* infection.

To understand the changes in metabolite levels and possible defense mechanisms of *A. muelleri*, we analyzed the metabolite profiles of *A. muelleri* corm samples (FC, FD, LC, LD). A total of 1,525 metabolites were detected in all samples. The results of subsequent model test and differential metabolite screening by orthogonal partial least squares discriminant analysis (OPLS-DA) revealed 21 significantly upregulated and 52 significantly downregulated metabolites in the LC vs LD comparison (**Figure 6A**) and 103 significantly upregulated and 27 significantly downregulated metabolites in the FC vs FD comparison (**Figure 6B**). In the FC vs FD and LC vs LD comparisons, the DAMs mainly included metabolites related to phenylpropane, amino acid, carbohydrate, lipid, phenolic acid, terpenoid, and alkaloid metabolism.

the DAMs mainly included phenylpropane metabolism pathway metabolites, amino acid metabolites, carbohydrate metabolites, lipid metabolites, phenolic acid metabolites, terpenoids, and alkaloids, among others. These results indicate that *F. solani* infection altered metabolite levels in *A. muelleri* corms.

**Figure 7:**
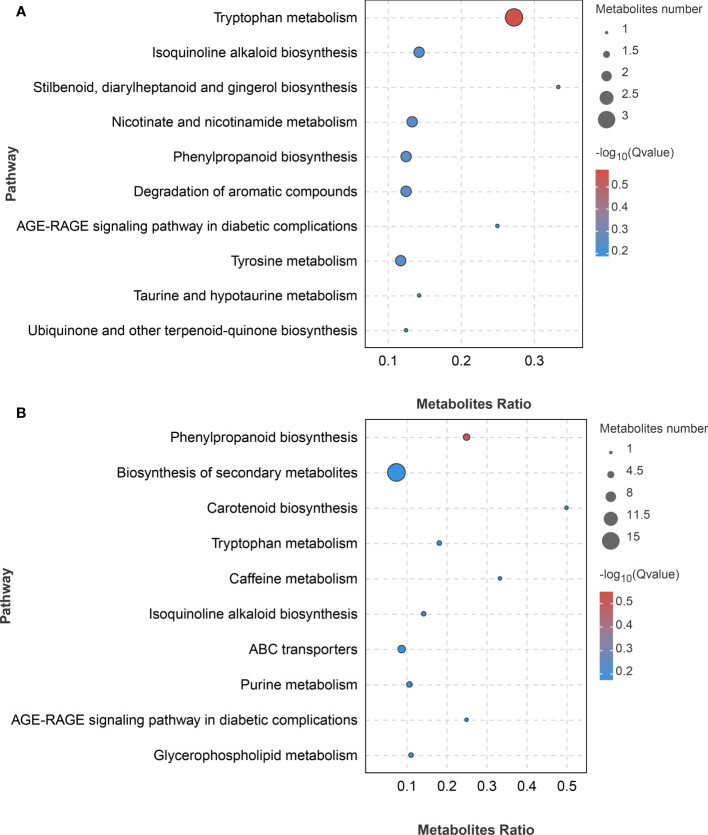
KEGG bubble diagram of DAMs identified in *F*. *solani*-infected leaf bud and flower bud corms of *A*. *muelleri*. **(A, B)** Leaf bud corms **(A)** and flower bud corms **(B)** of *A*. *muelleri* at 3 d after *F*. *solani* infection.

To identify the main pathways and metabolites used by *A. muelleri* to respond to *F. solani*, we performed the KEGG analysis of DAMs. The DAMs identified in the LC vs LD comparison were significantly enriched in ‘Tryptophan metabolism’, ‘Isoquinoline alkaloid biosynthesis’, ‘Stilbenoid, diarylheptanoid and gingerol biosynthesis’, ‘Nicotinate and nicotinamide metabolism’, and ‘Phenylpropanoid biosynthesis’, among other pathways (**Figure 7A**), while those identified in the FC vs FD comparison were significantly enriched in ‘Phenylpropanoid biosynthesis’, ‘Biosynthesis of secondary metabolites’, ‘Carotenoid biosynthesis’, and ‘Tryptophan metabolism’, among other pathways (**Figure 7B**). These results showed that metabolic pathways related to disease resistance were significantly enriched, indicating that *F. solani* infection activates the defense mechanism of *A. muelleri*.

### 3.4 Conjoint analysis

In biological systems, transcription and metabolism do not occur independently. Therefore, we analyzed the association between transcription and metabolism based on the theory that “genes or metabolites involved in the same biological process have the same or similar change patterns” (Cho et al., 2016). The conjoint KEGG enrichment analysis of DEGs and DAMs revealed 97 co-mapped pathways, including 35 and 38 co-mapped pathways in the FC vs FD and LC vs LD comparisons of DAMs, respectively (**Supplemental Tables 3**, **4**). Among these co-mapped pathways, ‘phenylpropanoid biosynthesis’, ‘isoquinoline alkaloid biosynthesis’ and ‘ubiquinone and other terpenoid-quinone biosynthesis’, among other secondary metabolite synthesis pathways, were common to both comparisons. This suggests that multiple secondary metabolic pathways are activated in *A. muelleri* corms in response to *F. solani* infection. The combined analysis of transcriptomics and metabonomic data showed that the O2PLS model was reliable (R^2^ > 0.75). Pearson correlation coefficients of the DEGs and DAMs were consistent. The absolute value of the correlation coefficient was greater than 0.5, and the top 250 DEGs and the corresponding metabolites were further selected and represented as a network diagram (**Supplemental Figure S3**).

### 3.5 Phenylpropanoid biosynthesis

**Figure 8:**
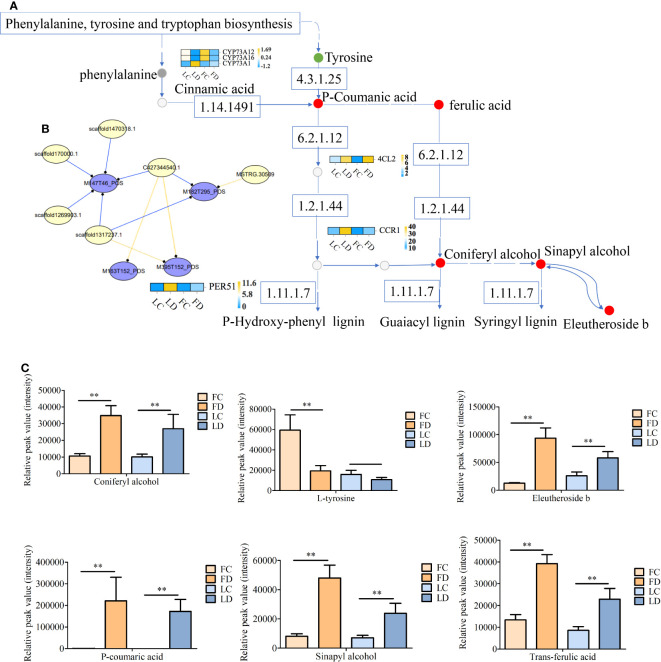
Schematic of the phenylpropane biosynthesis pathway. **(A, B)** Correlation diagram **(A)**, correlation network diagram of DEGs and DAMs **(B)**, and histogram of the relative content of DAMs involved in the phenylpropane biosynthesis pathway **(C)**. MSTRG.30509: 4-coumarate-CoA ligase (4CL2); scaffold1470318.1: peroxidase (PER51); C427344540.1: trans-cinnamate 4-monooxygenase (CYP73A12); scaffold1317237.1: trans-cinnamate 4-monooxygenase (CYP73A16); scaffold170000.1: trans-cinnamate 4-monooxygenase (CYP73A1); scaffold1269903.1: cinnamoyl-CoA reductase (CCR1); M182T295_POS: DL-tyrosine; M163T152_POS: coniferyl alcohol; M177T173_POS: trans-ferulic acid; M395T152_POS: eleutheroside b; M193T150_POS: sinapyl alcohol; M147T46_POS:P-coumaric acid. In **(B)**, yellow line indicates negative regulation, and blue line indicates positive regulation. ** on the bars indicate significant differences at different infection times (Student’s t-test, P < 0.05).

Plants employ the phenylpropanoid biosynthesis pathway in response to defense against pathogens. In this study, he phenylpropanoid biosynthesis pathway was significantly enriched in *F. solani*-infected *A. muelleri* corms. Key metabolites in the phenylpropanoid biosynthesis pathway, including L-tyrosine, p-coumaric acid, trans-FA, coniferyl alcohol, sinapyl alcohol, and eleutheroside b, exhibited different levels in different treatments; these compounds were increased by factors of -1.6, 9.24, 1.55, 1.72, 2.56, and 2.88, respectively, in FD samples compared with FC samples, and by factors of -0.56, 9.92, 1.41, 1.42, 1.76, and 1.16, respectively, in LD samples compared with LC samples (**Figure 8**). Moreover, key phenylpropanoid biosynthesis genes, including *AmPER51*, *AmCYP73As*, *Am4CL2*, and *AmCCR1*, were upregulated (**Figure 8**). These results indicate that the phenylpropanoid biosynthesis pathway is involved in the interaction between *A. muelleri* and *F. solani.*

### 3.6 Validation of candidate DEGs based on qRT-PCR analysis

**Figure 9:**
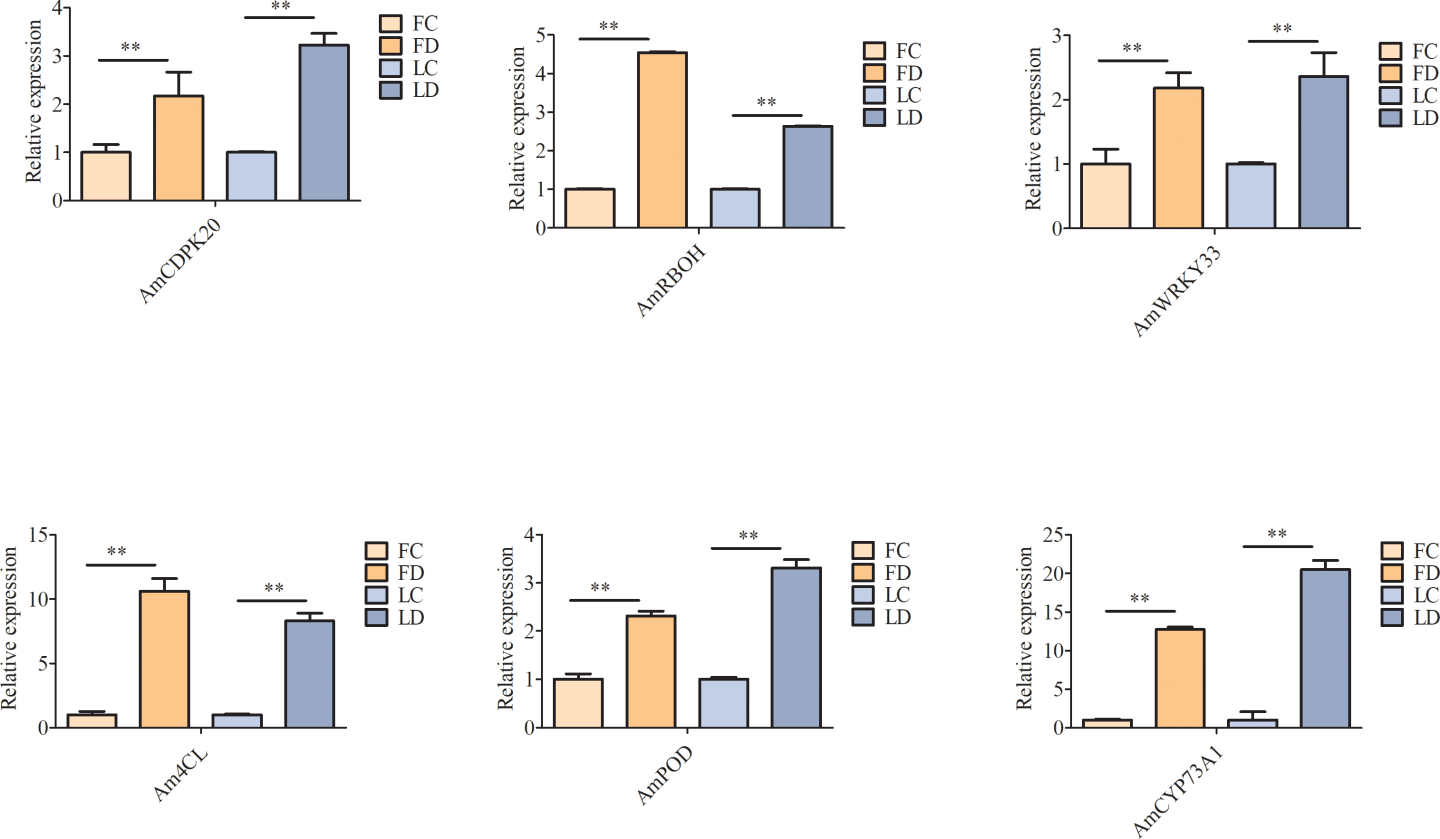
Verification of the expression profiles of six unigenes by qRT-PCR. ** on the bars indicate significant differences at different infection times (Student’s t-test, P < 0.05).

To determine whether the candidate DEGs were involved in the *A. muelleri* response to *F. solani*, we screened the expression of six genes, including three ‘plant-pathogen interaction’ pathway genes (*AmCDPK20*, *AmRBOH*, and *AmWRKY33*) and three ‘phenylpropane biosynthesis’ pathway genes (*Am4CL*, *AmCYP73A1*, and *AmPOD*), by qRT-PCR. The results showed that all six genes were significantly upregulated in *A. muelleri* after infection with *F. solani* (**Figure 9**), indicating that these six genes could be used as candidates to study the response of konjac to *F. solani* in future studies.

### 3.7 Inhibition of *Fusarium solani* growth by FA treatment

**Figure 10:**
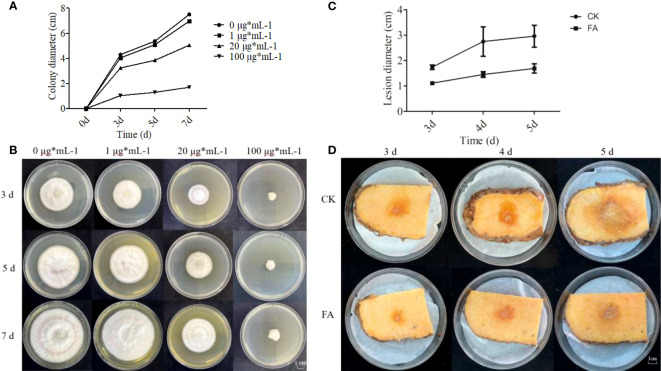
Effects of different concentrations of ferulic acid (FA) on the growth of *F. solani* and *A. muelleri*. **(A–D)** Effects of FA on the colony diameter **(A)** and strain growth **(B)** of *F. solani*, and on the lesion diameter **(C)** and the resistance phenotype **(D)** of *F. solani*-inoculated *A. muelleri* corms.

FA is an intermediate product of the phenylpropane biosynthesis pathway. To verify whether the phenylpropane biosynthesis pathway is involved in the defense of konjac corms against *F. solani*, *in vitro* antifungal tests against *F. solani* were conducted in the presence of FA. The results showed that as the concentration of FA increased, the inhibition rate of *F. solani* mycelium gradually increased; the inhibition rate was 15.64% at 1 μg mL^-1^ FA, 28.94% at 20 μg mL^-1^ FA, and 54.6% (approaching half of the maximum effective concentration) at 100 μg mL^-1^ FA (**Figure 10A**). Moreover, the growth rate of the control group was higher than that of the other FA treatment groups, and the colonies of the control group filled almost the entire culture dish by the 7th day of cultivation (**Figure 10B**). The FA treatment groups showed inhibitory effects on the growth of colonies, and the inhibitory effect increased with the increase in FA concentration (**Figure 10C**). These results indicate a positive correlation between the concentration of FA and the inhibition rate of *F. solani* mycelial growth.

### 3.8 Exogenous FA treatment of *Amorphophallus muelleri* corms

To test whether FA enhances the resistance of *A. muelleri* to *F. solani*, resistance experiments were conducted at FA concentration of 100 μg mL^-1^, based on the inhibitory effect of different concentrations of FA. In the FA treatment group, the lesion diameters on *A. muelleri* corms were 1.11, 1.45, and 1.69 cm at 3, 4, and 5 dpi, respectively; however, in the control group, the lesion diameters were 1.77, 2.32, and 2.77 cm, respectively (**Figure 10D**). The lesion diameter in the FA treatment group was significantly smaller than that in the control group. This indicates that exogenous FA treatment can effectively enhance the resistance of konjac to *F. solani*.

## 4 Discussion

### 4.1 RNA-Seq analysis of *Amorphophallus muelleri* resistance to *Fusarium solani*

After infection with *F. solani*, *A. muelleri* exhibits reduced yields and poor-quality corms, which affect the growth of seedlings, causing serious economic losses to farmers and producers. With advancements in next-generation sequencing, RNA-Seq has become an important means for researchers to study the mechanism of plant disease resistance. Through physiological index determination and transcriptome sequencing, Peng et al. (2022) showed that the expression of multiple core defense genes involved in fungal disease resistance and hormone signaling pathways is induced in tomato (*Solanum lycopersicum*) upon *Cladosporium fulvum* infection. Wei et al. (2022) sequenced the transcriptome of *Amorphophallus* spp. with high resistance or susceptibility to *Pectobacterium carotovorum* subsp. *carotovorum*, and showed that genes involved in plant hormone signaling, phenylpropane synthesis, and plant–pathogenic interactions regulate the response of *Amorphophallus* spp. to *P. carotovorum*. Liu et al. (2019) performed a transcriptome analysis and noted that the synergistic effect of jasmonic acid and ethylene signaling positively regulates the defense response of *Panax notoginseng* to *F. solani.* In this study, numerous DEGs and DAMs were identified in the flower bud and leaf bud corms of *A. muelleri* corms upon *F. solani* infection. Several DEGs were related to various defense responses to *F. solani*, including those involved in ‘plant-pathogenic interaction’, ‘mitogen-activated protein kinases (MAPKs)’, ‘plant hormone synthesis and signal transduction’, and ‘phenylpropane biosynthesis’. Upon infection by *F. solani*, the pattern recognition receptors (PRRs) of *A. muelleri* bind to pathogen-associated molecular patterns (PAMPs) to form early immune system warning signals and stimulate disease resistance reactions, including the induction of antioxidant burst enzyme genes (*AmRBOHD*), Ca^2+^ channel related genes (*AmCALM*), and MAPK signaling pathway transduction genes (*AmMAPKs*). Additionally, to prevent *F. solani* infection, *A. muelleri* plants express resistance protein-encoding genes (*AmFLS2* and *AmPTI*) and molecular chaperone gene (*AmHtpG*), which leads to a hypersensitive response (HR) at the infection site, promoting cell death. This indicates that *F. solani* infection induces effector-triggered immunity in *A. muelleri*. Together, these transcriptomic data suggest that multiple metabolic activities are associated with the defense of in *A. muelleri* against *F. solani*, which is consistent with the fact that the plants have evolved complex defense mechanisms during their interactions with pathogenic bacteria (Nobori and Tsuda, 2019; Teixeira et al., 2019).

### 4.2 Metabolomics analysis of *Amorphophallus muelleri* resistance to *Fusarium solani*

The biosynthesis of secondary metabolites is an important strategy employed by plants to defend against pathogen infections. Infection by pathogenic bacteria induces the synthesis of secondary metabolites, such as terpenes and alkaloids, in plants, which enhance plant immunity (Zaynab et al., 2018; Marone et al., 2022). In wheat (*Triticum aestivum*), Farahbakhsh et al. (2019) employed UPLC-QTOF/MS to compare the metabolic changes induced by wheat streak mosaic virus (WSMV) inoculation in highly resistant and susceptible varieties, and found that amino acid metabolism, lipid metabolism, and alkaloid metabolism pathways are involved in the defense response of wheat to WSMV. Similarly, Khizar et al. (2020) used UPLC-QTOF/MS to detect the differences in metabolite accumulation between *Aspergillus tubingensis*-resistant and -susceptible varieties of cotton (*Gossypium hirsutum*), and found that phenylpropanoids (stilbenes and furanocoumarin), flavonoids (phlorizin and kaempferol), alkaloids (indolizine and acetylcorynoline), and terpenoids (azelaic acid and oleanolic acid) accumulated to higher levels in resistant varieties than in susceptible varieties. Under *F. solani* stress, the level of 115 secondary metabolites was significantly increased in the flower bud and leaf bud corms of *A. muelleri*. These DAMs were mainly involved in ‘phenylpropane biosynthesis’, ‘arachidonic acid metabolism’, ‘stilbene, diarylheptane and gingerolin biosynthesis’, ‘isoquinoline alkaloids biosynthesis’, and other disease resistance-related metabolic pathways. In addition, the contents of 73 secondary metabolites were significantly decreased in *A. muelleri* flower bud corms and leaf bud corms under *F. solani* stress. These DAMs were mainly involved in ‘glycolysis/gluconeogenesis’, ‘purine metabolism’, and ‘fructose and mannose metabolism’. These results showed that when infected by *F. solani*, the contents of disease resistant metabolites increased in *A. muelleri* corms, inhibiting respiratory activity. The results of this study are consistent with the inhibition of respiration in *Actinidia chinensis* and *Capsicum annuum* after infection with *Pseudomonas syringae* pv. *actinidiae* and *Phytophthora capsica*, respectively (Stamler et al., 2015; Li et al., 2020).

### 4.3 Role of phenylpropane biosynthesis pathway in *Fusarium solani* resistance

The phenylpropane biosynthesis pathway, one of the critical secondary metabolic pathways of plants, and its metabolites, such as lignin, phenols, isoflavones, and organic acids, play an important role in regulating plant disease resistance, growth, and development. Chen et al. (2021) found that *Pichia galeiformis* improved the resistance of *Citrus reticulata* to *Penicillium digitatum* by upregulating the expression of key phenylpropane biosynthesis genes, enhancing the activity of key enzymes such as phenylalanine ammonia lyase (PAL) and 4-coumarate-CoA ligase (4CL), and increasing the content of total phenols, flavonoids, and lignin. Qu et al. (2022) showed that melatonin enhanced the postharvest disease resistance of *Vaccinium* sp. through enhancing the activity of PAL, 4CL, and other enzymes related to phenylpropane biosynthesis, improving the expression of genes related to phenylpropane metabolism,and promoting the accumulation of total phenols, flavonoids, anthocyanins, and lignin. In this study, the flower bud and leaf bud corms of *A. muelleri* were inoculated with *F. solani*, and transcriptome analysis revealed the upregulation of phenylpropane biosynthesis genes, including *Am4CL*, *AmCYP73A1*, *Am PAO5*, *AmPER51*, and *AmCCR1*. metabolome analysis showed that the level of L-tyrosine decreased, while the level of coniferyl alcohol, trans-FA, eleutheroside b, sinapyl alcohol, and p-coumaric acid increased. Moreover, the combined analysis of transcriptomics and metabolomics data also showed the enrichment of the phenylpropane biosynthesis pathway and a strong correlation between the phenylpropane biosynthesis related DEGs and DAMs. This result indicates that the phenylpropane biosynthesis pathway is involved in the defense of *A. muelleri* corms against *F. solani*.

FA is an important compound in the phenylpropane biosynthesis pathway. The effects of FA on *Fusarium* spp. have been explored in several studies. Martínez-Fraca et al. (2022) determined that the content of FA in the seeds of *Fusarium verticillioides* resistant maize varieties was significantly higher than that in the seeds of susceptible varieties. *In vitro* treatment with high-concentration FA extract inhibited the growth of fungi, and its effect was equivalent to FA ranging in concentration from 0.25–0.5 mM. Ponts et al. (2011) tested five phenolic acids (ferulic, coumaric, caffeic, syringic, and p-hydroxybenzoic acids) in radial growth assays, and found that FA exhibited the strongest antifungal activity against *Fusarium graminearum.* Consistent with the above studies, we found that 100 μg mL^-1^ FA was the most effective against *F. solani*, and the inhibitory effect on the growth of *F. solani* mycelium increased with the increase in FA concentration. Besides, Zheng et al. (2019) showed that the content of free FA, p-coumaric acid and conjugated t-cinnamic acid in *Brassica napus* is an important factor affecting cultivar resistance to *Verticillium longisporum*. Patzke and Schieber (2018) tested five phenolic acids (phlorizin, resveratrol, FA, 5-N-alkylresorcinols, and quercetin) for their ability to inhibit fungal growth, and found that FA was highly effective against *Botrytis cinerea*. Zhang et al. (2022) pointed out that FA enhances the ability of poplar trees to limit the growth of *Colletotrichum gloeosporioides*. Similarly, in this study, exogenous FA treatment significantly enhanced the resistance of *A. muelleri* corms to *F. solani*.

## 5 Conclusion

In conclusion, this study combined RNA-Seq and metabolome analyses to investigate the defense response of *A. muelleri* corms to *F. solani* infection. Under *F. solani* stress, *A. muelleri* leaf bud corms and flower bud corms exhibited similar disease resistance patterns. A series of plant disease resistance related pathways were activated, including ‘plant hormone signal transduction’, ‘MAPK cascade reaction’, ‘plant-pathogen interaction’, and ‘phenylpropane biosynthesis’. Moreover, *AmCDPK20*, *AmRBOH*, *AmWRKY33*, *Am4CL*, *AmPOD*, and *AmCYP73A1* appeared to participate in the defense against *F. solani.* In addition, according to metabolomic data analysis, secondary metabolite biosynthesis pathways, including ‘phenylpropane biosynthesis’, ‘arachidonic acid metabolism’, ‘stilbene, diarylheptane and gingerol biosynthesis’, and ‘isoquinoline alkaloid biosynthesis’, were induced upon *F. solani* infection. Finally, the conjoint analysis of DEGs and DAMs revealed that the phenylpropane biosynthesis pathway plays a very important role in the defense response of *A. muelleri* corms against *F. solani* infection. Notably, FA treatment inhibited the growth of *F. solani* and alleviated the disease symptoms on *A. muelleri* corms. This study not only provides a list of candidate genes and metabolites involved in *A. muelleri* defense against *F. solani* but also lays a strong foundation for further investigation of the molecular mechanism of *A. muelleri* resistance to *F. solani*.

## Data availability statement

The datasets presented in this study can be found in online repositories. The names of the repository/repositories and accession number(s) can be found in the article/**Supplementary Material**.

## Author contributions

PG, FH, and LY conceptualized this study and executed the experiments; YQ, LL, and SY provided experimental guidance; HW and JL participated in processing the data and writing the manuscript. All authors contributed to the article and approved the submitted version.

## Supplementary material

Supplementary Figure 1 Morphological evaluation of *F. solani* cultured on PDA medium for 7 days. **(A, B)** Morphology of the culture **(A)** and of hyphae and conidia **(B)**.

Supplementary Figure 2 Principal component analysis (PCA) of the transcriptomic changes in infected and healthy *A. muelleri* corms. LC, disease-free leaf bud corms; LD, leaf bud corms inoculated with *F. solani* for three days; FC, disease-free flower bud corms; FD, flower bud corms inoculated with *F. solani* for three days.

Supplementary Figure 3 Heatmap of the top 250 DEGs and the corresponding metabolites.

Supplementary Table 1 Primers used for qPCR.

Supplementary Table 2 Statistics of the RNA-Seq data of *A. muelleri* corms infected with *F. solani*.

Supplementary Table 3 Conjoint enrichment analysis of the DEGs and DAMs identified between healthy and infected *A. muelleri* flower bud corms.

Supplementary Table 4 Conjoint enrichment analysis of the DEGs and DAMs identified between healthy and infected *A. muelleri* leaf bud corms.
