# Supplementary figures and images for: Amorphophallus muelleri activates ferulic acid and phenylpropane biosynthesis pathways to defend against Fusarium solani infection
Source: Front Plant Sci. 2023 Jul 5;14:1207970. doi: 10.3389/fpls.2023.1207970 (PMC10354422; doi:10.3389/fpls.2023.1207970)

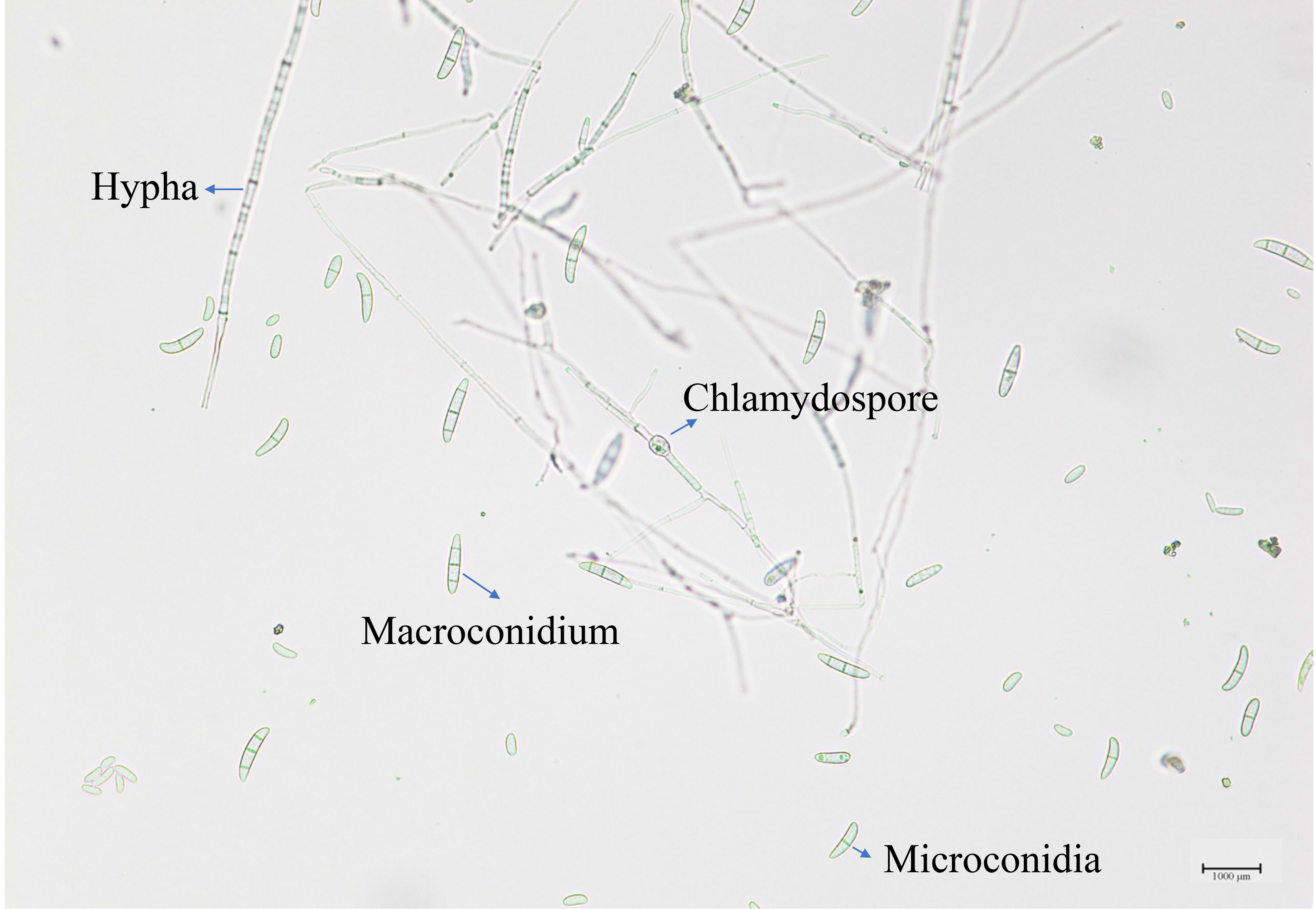

Supplement: Supplementary Figure 1 — Morphological evaluation of F. solani cultured on PDA medium for 7 days. (A, B) Morphology of the culture (A) and of hyphae and conidia (B). [file Image_1.tif]

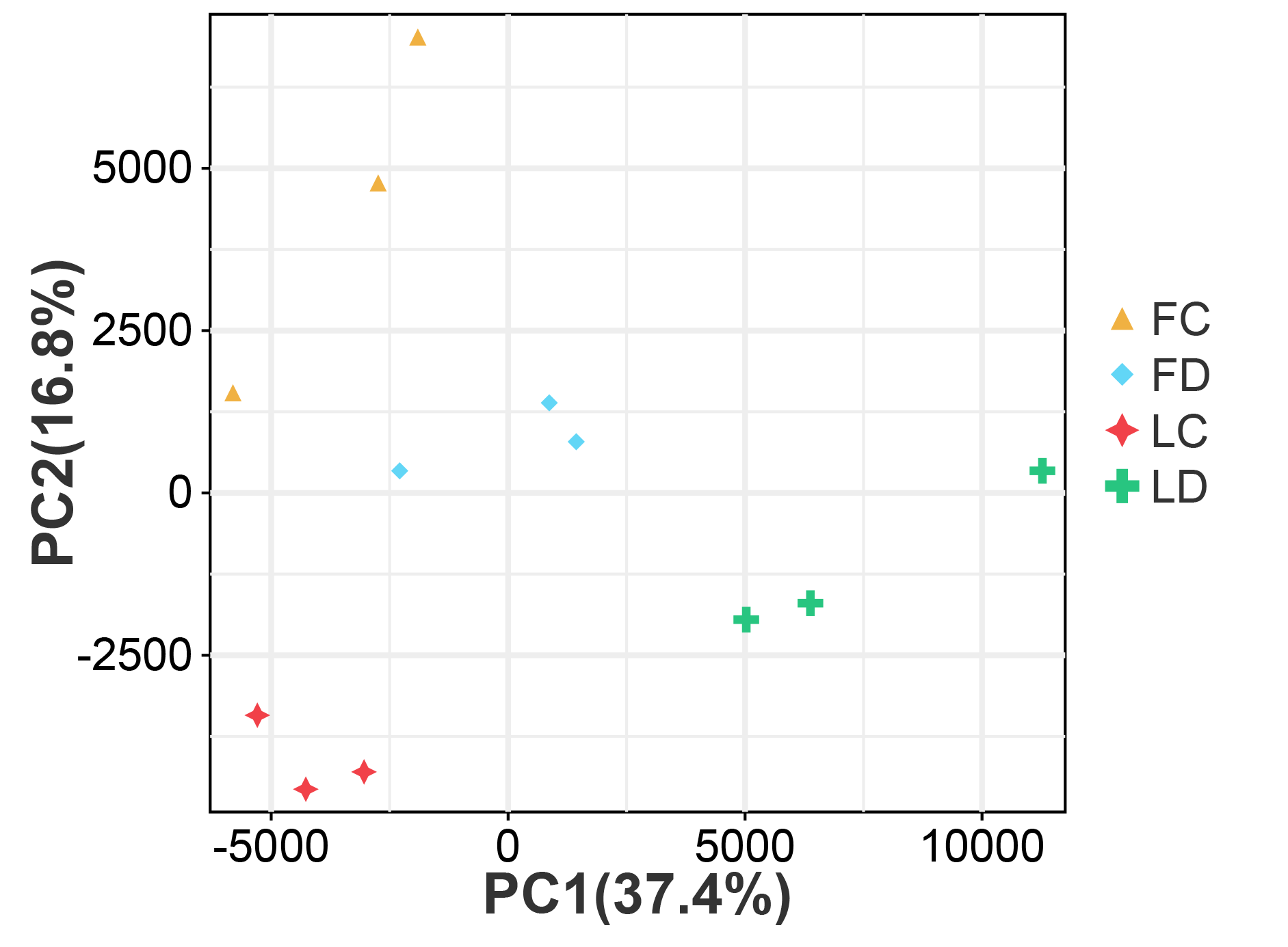

Supplement: Supplementary Figure 2 — Principal component analysis (PCA) of the transcriptomic changes in infected and healthy A. muelleri corms. LC, disease-free leaf bud corms; LD, leaf bud corms inoculated with F. solani for three days; FC, disease-free flower bud corms; FD, flower bud corms inoculated with F. solani for three days. [file Image_2.tif]

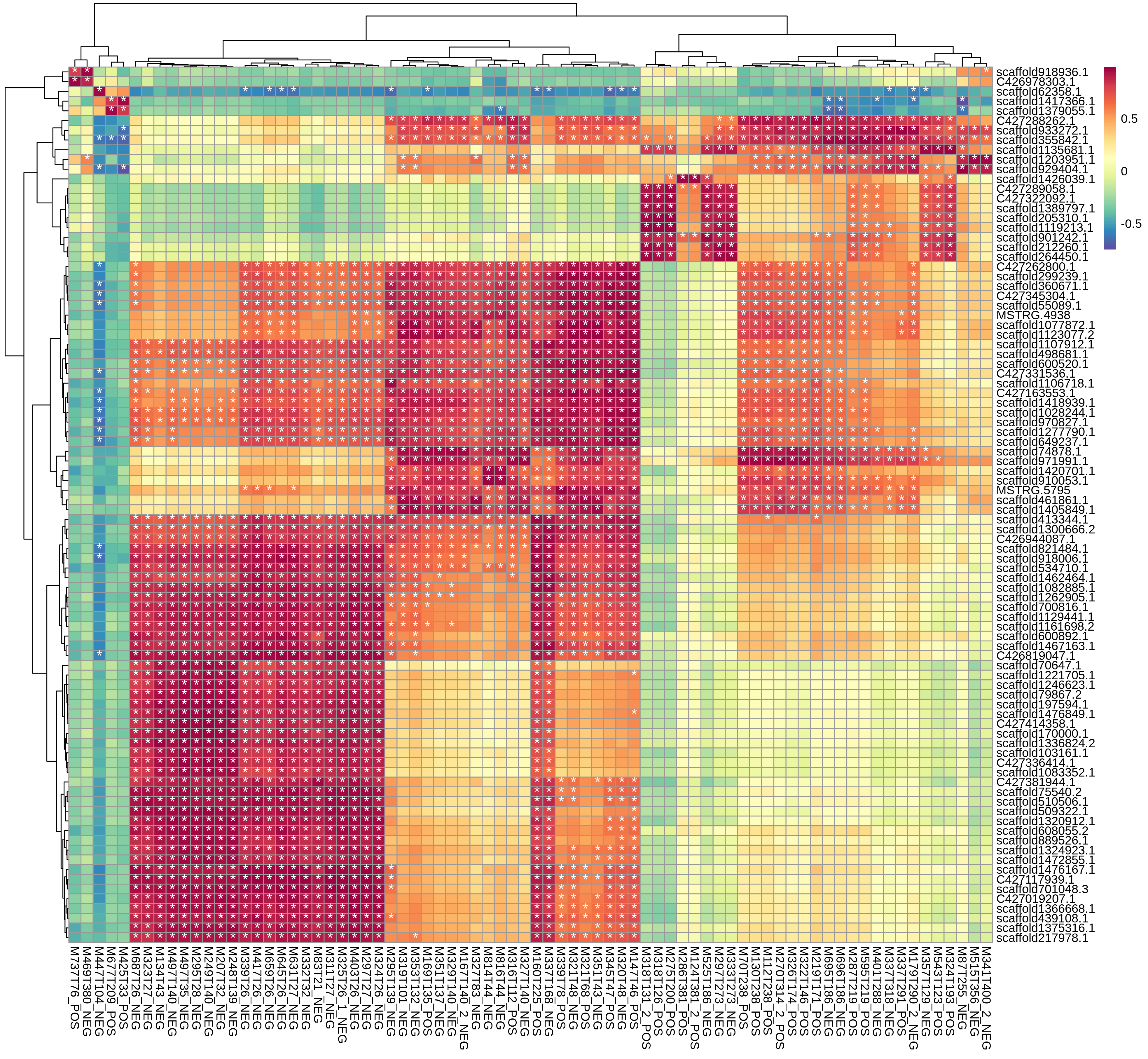

Supplement: Supplementary Figure 3 — Heatmap of the top 250 DEGs and the corresponding metabolites. [file Image_3.tif]
